# Chronic Lithium Treatment Alters NMDA and AMPA Receptor Synaptic Availability and Dendritic Spine Organization in the Rat Hippocampus

**DOI:** 10.2174/1570159X21666230913144420

**Published:** 2023-09-15

**Authors:** Lucia Caffino, Giorgia Targa, Anne Stephanie Mallien, Francesca Mottarlini, Beatrice Rizzi, Judith R. Homberg, Peter Gass, Fabio Fumagalli

**Affiliations:** 1 Department of Pharmacological and Biomolecular Sciences ‘Rodolfo Paoletti’, Università degli Studi di Milano, Via Balzaretti 9, 20133, Milan, Italy;; 2 Department of Psychiatry and Psychotherapy, RG Animal Models in Psychiatry, Central Institute of Mental Health, Medical Faculty Mannheim, Heidelberg University, Mannheim, Germany;; 3 Department of Cognitive Neuroscience, Division of Molecular Neurogenetics, Donders Institute for Brain, Cognition and Behaviour, Radboud University Nijmegen Medical Centre, The Netherlands

**Keywords:** Bipolar disorder, lithium, glutamate, NMDA receptor, AMPA receptor, hippocampus, BDNF

## Abstract

**Background:**

The mechanisms underlying the action of lithium (LiCl) in bipolar disorder (BD) are still far from being completely understood. Previous evidence has revealed that BD is characterized by glutamate hyperexcitability, suggesting that LiCl may act, at least partially, by toning down glutamatergic signaling abnormalities.

**Objective:**

In this study, taking advantage of western blot and confocal microscopy, we used a combination of integrative molecular and morphological approaches in rats exposed to repeated administration of LiCl at a therapeutic dose (between 0.6 and 1.2 mmol/l) and sacrificed at two different time points, *i.e*., 24 hours and 7 days after the last exposure.

**Results:**

We report that repeated LiCl treatment activates multiple, parallel, but also converging forms of compensatory neuroplasticity related to glutamatergic signaling. More specifically, LiCl promoted a wave of neuroplasticity in the hippocampus, involving the synaptic recruitment of GluN2A-containing NMDA receptors, GluA1-containing AMPA receptors, and the neurotrophin BDNF that are indicative of a more plastic spine. The latter is evidenced by morphological analyses showing changes in dendritic spine morphology, such as increased length and head diameter of such spines. These changes may counteract the potentially negative extra-synaptic movements of GluN2B-containing NMDA receptors as well as the increase in the formation of GluA2-lacking Ca^2+^-permeable AMPA receptors.

**Conclusion:**

Our findings highlight a previously unknown cohesive picture of the glutamatergic implications of LiCl action that persist long after the end of its administration, revealing for the first time a profound and persistent reorganization of the glutamatergic postsynaptic density receptor composition and structure.

## 1 INTRODUCTION

Bipolar disorder (BD) is a common, chronic psychiatric disorder, often life-threatening, with high prevalence characterized by shifts between depressive and manic episodes [1].

Its underlying etiology and neurobiology are still obscure, whereas the disorder's pathophysiology has been demonstrated to involve functional and structural alterations in neuronal plasticity, as shown in BD patients [2].

Lithium (LiCl) is an alkali metal historically used as a mood stabilizer, with an unknown role in human physiology. In fact, despite its wide use for treating BD and the fact that it is still the first-line medication [3], the mechanisms underlying its actions are not yet fully understood [4]. Recently, the hippocampus, a brain area involved in learning and memory processes, has been proposed as the main target of LiCl therapeutic mechanism of action: in both human and animal models, LiCl accumulates in the hippocampal regions, thus normalizing its volume [5-7]. Moreover, several mechanisms have been put forward to explain LiCl efficacy, ranging from alterations of monoaminergic neurotransmitter systems to activation of intracellular signaling pathways or alteration of neuroplastic mechanisms, calling for a non-pharmacological selectivity of this drug.

Recently, it has been suggested that BD is characterized by glutamate hyperexcitability. In fact, the activity of the glutamatergic network appears to be overactive, mainly in the manic phase of BD [8, 9]. Furthermore, high levels of glutamate have been found in the brain of BD patients [10, 11]. The involvement of glutamate has been further substantiated by experimental evidence showing that LiCl acts, at least in part, by toning down the hyperactivity of the glutamatergic system. In fact, Mertens and co-workers (2015) developed an iPSC model for BP, observing a hyperexcitability phenotype that responds to LiCl [12]; additionally, Nonaka and colleagues (1998) have shown that long-term LiCl treatment protects hippocampal neurons against glutamate excitotoxicity involving NMDA-mediated apoptosis [13] further pointing to glutamate receptor hyperactivity as a culprit of this disorder. Recent data pointing to the neurotrophin BDNF as pivotal for LiCl antimanic effects by reducing AMPA excitatory postsynaptic currents in the hippocampus [14] further indicate LiCl's complex mechanism of action. Indeed, recent evidence has suggested that LiCl may also reduce the presynaptic release of glutamate, dampening excitatory neurotransmission [15, 16], an effect that may counteract the increased release of glutamate previously observed [17, 18]. To sum up, these lines of evidence draw a cohesive picture indicating that one of LiCl's features is the ability to counterbalance glutamate hyperactivity.

Several preclinical investigations have shown that various glutamatergic functions are affected by chronic LiCl treatment, despite some variability across experiments [13, 19-21]. Accordingly, we hypothesize that LiCl exerts its therapeutic activity by restoring glutamate homeostasis *via* structural and functional reorganization of the glutamate synapse. Thus, the main objective of the present manuscript was to study the complex machinery that regulates the glutamate synapse in the hippocampus following repeated exposure to LiCl. Accordingly, we set out to evaluate the expression of critical glutamatergic determinants in different cellular preparations, *i.e*., whole homogenate, post-synaptic density, and extra-synaptic compartments, to distinguish between the effects of LiCl on the translation of glutamatergic proteins and their trafficking between synaptic and extra-synaptic space.

Further, molecular assessments of glutamate determinants were performed at two different time points, *i.e*., 24 hours (*i.e*., under a LiCl -free state) and 7 days (to investigate the drug's long-term effect) following the last exposure. We are aware that while these times of sacrifice may not inform us on the onset of molecular alterations, they could, however, instruct on the long-lasting nature of such alterations. We evaluated the expression of the glial glutamate transporter (GLT-1) regulating the neurotransmitter reuptake [22] and of xCT, the light chain component of the system Xc-, a cystine/glutamate antiporter that mediates the exchange of extracellular cystine and intracellular glutamate across the cellular plasma membrane [23]. At the post-synaptic site, we investigated the expression of the main subunits of NMDA (GluN1, GluN2A, GluN2B) and AMPA (GluA1, GluA2, GluA3) receptors. Also, we measured the expression of integral proteins of the glutamate synapse, such as the postsynaptic density protein 95 (PSD95) [24] and of neuroligin-1, a trans-synaptic adhesion molecule that stabilizes pre- and post-synaptic excitatory boutons in an activity-dependent fashion [25]. In addition, apart from NMDA and AMPA ionotropic receptors, we also evaluated mGluR5, a metabotropic glutamate receptor that exhibits a wide range of modulatory and regulatory functions on other receptors, particularly on ionotropic glutamate receptors, and to be markedly reduced after LiCl exposure [21, 26]. Then, considering the effect of LiCl on glutamate-mediated calcium (Ca^2+^) response by altering various intracellular signaling cascades by decreasing calcium signaling, we measured the Ca^2+^/CaM-dependent protein kinase II type a (αCαMKII), an intracellular Ca^2+^ sensor, evaluating the levels of αCαMKII phosphorylation as an index of protein activation [27]. We have also studied the morphology of dendritic spines, examining their number and shape, often indicative of changes in the postsynaptic density composition and synaptic strength. Given the role of BDNF in modulating glutamatergic neurotransmission [28], we finally investigated the expression and activation of the BDNF-TrkB-dependent pathway.

## 2 MATERIALS AND METHODS

### 2.1 Animals

Subjects of this study were 52 male Wistar rats (Charles River Laboratories, Sulzfeld, Germany), which were eight weeks old (277.26 g ± 1.586 g body weight) on arrival. They were housed in groups of four in type 4 Macrolon cages with bedding (Abedd Espen MIDI, ABEDD, Vienna, Austria), tissue, wooden curl nesting material (sniff Spezialdiäten, Soest, Germany), cardboard tunnel (ssniff Spezialdiäten, Soest, Germany) and wooden sticks (espen bricks, sniff Spezialdiäten, Soest, Germany) in a room with 12 h/12 h light cycle (lights on 7 am) and settings to humidity 45% ± 5% and temperature 22 ± 2℃. Rats were fed a diet (Rat/Mouse Maintenance V1534, sniff Spezialdiäten, Soest, Germany) of 20 g per day (on weekends, 25 g per rat per day). Water was provided ad libitum. The animals were supplied with fresh water twice a week and new cages once per week. Body weight was assessed daily.

### 2.2 Treatment

Rats were randomly assigned to one of the two treatment groups Saline (Ctrl, NaCl) or Lithium Chloride (LiCl). They received a daily i.p. injection with either substance for 14 days.

We used a regime with increasing doses, as reported by Popovic *et al*. in 2019 [29]. LiCl was diluted in Saline (Sigma Aldrich, Germany). The initial dose was 1.5 mEq/kg for four days. Thereafter, animals received 2.3 mEq/kg for seven days and 3.0 mEq/kg for the last three days. The control group received a daily i.p. injection with 2 mL/kg saline solution. LiCl treatment resulted in 24 h nadir plasma levels of 0.4 ± 0.13 mmol/l, as analyzed in rats used for Experiment 2 (see below). These levels correspond to 12 h nadir plasma levels between 0.6 and 1.2 mmol/l aimed for antimanic therapy in humans [30]. Seven days later, LiCl treatment resulted in -0.04 ± 0.00 mmol/l.

#### 2.2.1 Experiment 1

A total of 36 rats were euthanized by decapitation either 24 hours or 7 days after the last injection (n = 9/group). Brains were removed, dissected on ice, and hippocampi were frozen on dry ice and stored at -80℃ until further analysis.

#### 2.2.2 Experiment 2

A total of 16 rats were anesthetized 24 hours or 7 days after the last injection (n = 4/group) with ketamine/xylazine and transcardially perfused with 4% paraformaldehyde (Carl Roth, Germany) in 0.1 M phosphate buffer (PBS) as described earlier [31]. The brains were removed and postfixed for 40 min, washed two times in PBS, transferred into a new tube with 0.1 M PBS plus 0.2% sodium azide (Sigma Aldrich, Germany), and stored at 4℃ until further analysis. Right before starting the perfusion, heart blood was taken and collected in EDTA tubes and sent to Labor Limbach (Heidelberg, Germany) to analyze LiCl blood levels. No rats were excluded from this study. The experimenters were blinded to the treatment group. Only for injection preparation purposes one person was unblinded. All experiments were approved by German animal welfare authorities (Regierungspräsidium Karlsruhe) and complied with the European Union (European Communities Council Directive 2010/63/EU).

### 2.3 Preparation of Protein Extracts and Western Blot Assays

Proteins in the whole homogenate, post-synaptic, extra-synaptic, and nuclear fractions were analyzed as previously described with minor modifications [32, 33]. Briefly, hippocampi from adult male Wistar rats (n = 36) were homogenized in a Teflon-glass potter in cold 0.32 M sucrose buffer pH 7.4 containing 1 mM HEPES, 1 mM MgCl2, 1 mM NaHCO_3_, and 0.1 mM PMSF, in the presence of commercial cocktails of protease (complete™ Protease Inhibitor Cocktail, Roche, Monza, Italy) and phosphatase (Sigma-Aldrich, Milan, Italy). An aliquot of each homogenate was then sonicated and stored at -20℃. The remaining homogenate was centrifuged at 800 g for 5 min; the resulting pellet (P1), corresponding to the nuclear fraction, was resuspended in a buffer (HEPES, 20 mM; dithiothreitol, 0.1 mM; EGTA, 0.1 mM) with protease and phosphatase inhibitors, whereas the obtained supernatant was then centrifuged at 13000 g for 15 min, obtaining a pellet. This pellet was resuspended in a buffer containing 75 mM KCl and 1% Triton X-100 and centrifuged at 100000 g for 1 h. The resulting supernatant indicated as Triton X-100 soluble fraction (TSF, extra-synaptic fraction), was stored at -20℃; the pellet, referred to as PSD or Triton X-100 insoluble fraction (TIF, post-synaptic density), was homogenized in a glass-glass potter in 20 mM HEPES, protease and phosphatase inhibitors and stored at -20℃ in the presence of glycerol 30%.

The total amount of proteins has been measured in the homogenate, P1, TIF, and TSF fractions using the Bradford Protein Assay procedure (Bio-Rad, Milan, Italy), using bovine serum albumin as the calibration standard. Equal amounts of proteins of the homogenate (10 μg), nuclear fraction (15 μg), TIF fraction (8 μg), and TSF fraction (50 μg) were run on a sodium dodecyl sulfate 8% or 14% polyacrylamide gel under reducing conditions and then electrophoretically transferred onto nitrocellulose membranes (GE Healthcare, Milan, Italy). The entire nitrocellulose blot was cut close to the molecular weight at which protein bands are expected to be detected, as suggested by their specific molecular weight and the information depicted in the datasheet of the antibody. Blot strips were blocked for 1 h at room temperature with I-Block solution (Life Technologies Italia, Italy) in TBS 0.1% Tween- 20 buffer and incubated with antibodies against the proteins of interest.

The conditions of the primary antibodies were the following: anti-GluN1 (1:1000, Cell Signaling Technology, cod. 5704, RRID: AB_1904067), anti-GluN2A (1:1000, Cell Signaling Technology, cod. 4205, RRID: AB_2112295), anti GluN2B (1:1000, Cell Signaling Technology, cod. 14544, RRID: AB_2798506), anti-GLT-1 (1:2000, Abcam, cod. ab41621, RRID: AB_941782), anti xCT (1:1000, Abcam, Cod. ab216876), anti mBDNF (1:500, Icosagen, cod. 327-100, RRID: AB_2927780), anti TrkB (1:1000, Cell Signaling Technologies, cod. 4603, RRID: AB_2155125), anti phospho- CREB(Ser133) (1:1000, Cell Signaling Technologies, cod. 9198, RRID: AB_2561044), anti CREB (1:1000, Cell Signaling Technologies, cod. 9197, RRID: AB_331277), anti- GluA1 (1:1000, Cell Signaling Technology, cod. 13185, RRID: AB_2732897), anti-GluA2 (1:1000, Cell Signaling Technology, cod. 5306, RRID: AB_10622024), anti-GluA3 (1:1000, Millipore, cod. MAB5416, RRID: AB_2113897), anti phospho-αCαMKII (Thr286) (1:1000, Thermo Fisher Scientific, cod. MA1047, RRID: AB_325402), anti αCαMKII (1:2000, Millipore, cod. 05-532, RRID: AB_309787), anti mGluR5 (1:1000, Millipore, cod. Ab5675, RRID: AB_2295173), anti-PSD95 (1:2000, Cell Signaling Technology cod.3450, RRID: AB_2292883), anti- Neuroligin-1 (1:1000, Synaptic System, cod. 129 003, RRID: AB_887746) and anti β-actin (1:5000, Sigma-Aldrich, cod. A5441, RRID: AB_476744). The secondary antibodies used were goat anti-mouse (Sigma-Aldrich, cod. A4416, RRID: AB_ 258167) and goat anti-rabbit (Thermo Fisher Scientific, cod. 31460, RRID: AB_228341). Results were standardized using β-actin as the control protein, identified by evaluating the band density at 43 kDa. Immunocomplexes were detected by chemiluminescence using the Chemidoc MP Imaging System (Bio-Rad Laboratories, RRID: SCR_019037) and subsequently analyzed with Image LabTM software (Bio- Rad, RRID: SCR_014210).

Gels were run 2 times each, and the results represent the average from 2 different western blots, which were averaged and normalized by using a specific correction factor: Correction factor gel B = average of (OD protein of interest/ OD β-actin for each sample loaded in gel A)/(OD protein of interest/OD β-actin for the same sample loaded in gel B) [34]. Cropped immunoblots related to the protein expression levels measured in the whole homogenate, post-synaptic and extra-synaptic density and the nuclear fraction of hippocampi are presented in Supplementary Figs. (**1-7**).

### 2.4 Dendritic Spine Labeling and Morphological Classification

Neuronal labeling and morphological classification of dendritic spines in the whole hippocampus, primarily formed by pyramidal and granule cells, were achieved using the lipophilic membrane tracer 1,1’-Dioctadecyl- 3,3,3’,3’- Tetramethylindocarbocyanine Perchlorate (DilC18(3)) (Life Technologies), as previously published [35, 36]. The number of neurons used for quantification was at least 20 for each experimental group (from each neuron, a different number of dendritic segments was analyzed); the neurons analyzed were belonging to 8 hemispheres per group. The average dendritic length analyzed is 50 μm, and the length of the total dendrites analyzed was 1000 μm for each experimental group. Analysis of dendritic spine morphology was performed with Fiji software (RRID: SCR_002285) released by ImageJ software; each protrusion was manually traced and, specifically, spine length, head (Wh), and neck width (Wn) were measured. These parameters were used to classify and sort dendritic spines into three categories (thin, stubby, and mushroom). The classification scheme is as follows: protrusions with a length greater than 3 μm were categorized as filopodia, while those with a length below this threshold were classified as dendritic spines. Among the dendritic spines, those with a Wh/Wn ratio bigger than 1.7 were categorized as mushrooms, whereas those with a Wh/Wn ratio smaller than 1.7 were further subdivided into two groups: stubby if their length was less than 1 μm, and thin if their length exceeded 1 μm [37, 38]. An operator who was ‘blind’ to the experimental conditions performed both image acquisition and quantification.

### 2.5 Statistical Analysis

Data were collected in individual animals (independent determinations) and are presented as means and standard errors. Molecular and morphological changes produced by LiCl treatment were evaluated for the normality of residuals with the Kolmogorov-Smirnov test. Data with normal distribution were analyzed by unpaired Student’s t-test, using as control condition NaCl treated animals and as testing condition LiCl treated rats. Data with a non-normal distribution were analyzed by the Mann-Whitney test (U). Subjects were eliminated from the final dataset if their data deviated from the mean by 2 SDs. Prism 9 (GraphPad Software Prism v9, San Diego, CA, USA, RRID: SCR_002798) was used to analyze all data. Significance for all tests was assumed at *p <* 0.05.

## 3 RESULTS

**Fig. (1):**
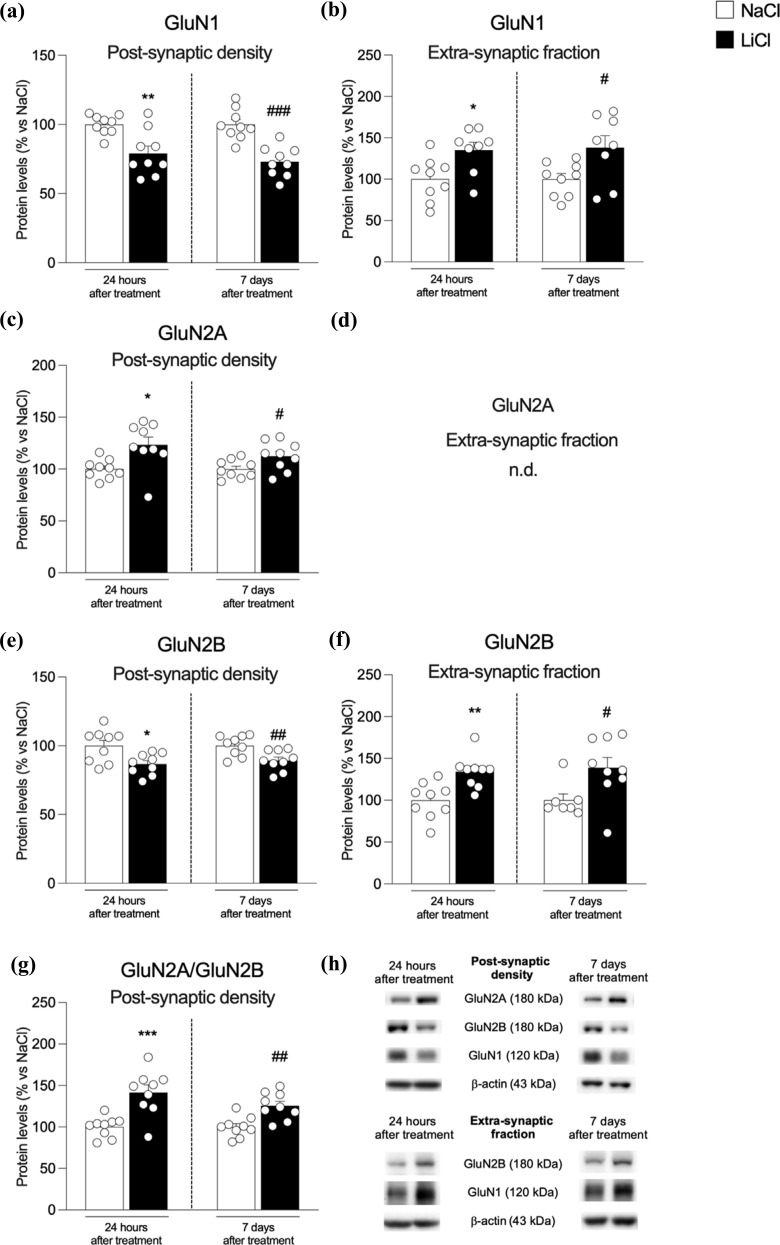
Protein expression levels of the main NMDA receptor subunits in the hippocampus of rats 24 h and 7 days after the last NaCl and LiCl injection. GluN1 (panel **a**, **b**), GluN2A (panel **c**, **d**), and GluN2B (panel **e**, **f**) levels were investigated in the postsynaptic density and the extra-synaptic fraction. The ratio GluN2A/GluN2B (panel **g**) was measured in the postsynaptic density. In panel (**h**), representative immunoblots for GluN1, GluN2A, GluN2B, and β-actin are shown for each fraction evaluated. Results are expressed as mean percentage ± mean standard error. Unpaired Student’s t-test *p < 0.05, **p < 0.01, ***p < 0.001 *vs.* NaCl 24 h; ^#^*p <* 0.05, ^##^*p <* 0.01, ^###^*p <* 0.001 *vs.* NaCl 7 d.

We first investigated the expression of NMDA receptor subunits in the post-synaptic density and the extra-synaptic fraction at two different time points, *i.e*., 24 hours or 7 days after the last exposure to LiCl (Fig. **1a**-**h**). The expression of the main subunit of NMDA receptors, GluN1, is significantly reduced at both time points in the post-synaptic density (Fig. **1a**: -21% *vs*. NaCl 24 h *p* = 0.002, t_(16)_ = 3.6 and -27% *vs*. NaCl 7 d, *p <* 0.0001, t_(16)_ = 5.24) but increased at extra-synaptic sites (Fig. **1b**: +35% *vs*. NaCl 24 h *p* = 0.015, t_(15)_ = 2.74 and +38% *vs*. NaCl 7 d, *p* = 0.025, t_(15)_ = 2.48). The analysis of the accessory subunits of NMDA receptor GluN2A and GluN2B revealed significant differences. In fact, we found increased expression of GluN2A at both 24 hours and 7 days post-LiCl treatment in the post-synaptic density (Fig. **1c**: +23% *vs.* NaCl 24 h *p* = 0.01, t_(16)_ = 2.9 and +12% *vs.* NaCl 7 d, *p* = 0.038, t_(16)_ = 2.25) while this subunit was absent at extra-synaptic sites, as previously shown [39]. Conversely, the expression of the GluN2B subunit follows the pattern observed for GluN1, with a significant reduction in the post-synaptic density (Fig. **1e**: - 13% *vs.* NaCl 24 h *p* = 0.011, t_(16)_ = 2.84 and -11% *vs.* NaCl 7 d, *p* = 0.007, t_(16)_ = 3.09) accompanied by a significant up- regulation at extra-synaptic sites (Fig. **1f**: +34% *vs.* NaCl 24 h *p* = 0.002, t_(16)_ = 3.56 and +39% *vs.* NaCl 7d, *p* = 0.025, t_(14)_ = 2.50). We then measured the ratio GluN2A/GluN2B, known to be considered an index of plasticity of synapses [40], and found it increased at both time points in the hippocampus of LiCl-treated rats Fig. **1g**: +42% *vs*. NaCl 24 h *p* = 0.0006, t_(16)_ = 4.23 and +26% *vs*. NaCl 7d, *p* = 0.001, t_(16)_ = 3.80).

**Fig. (2):**
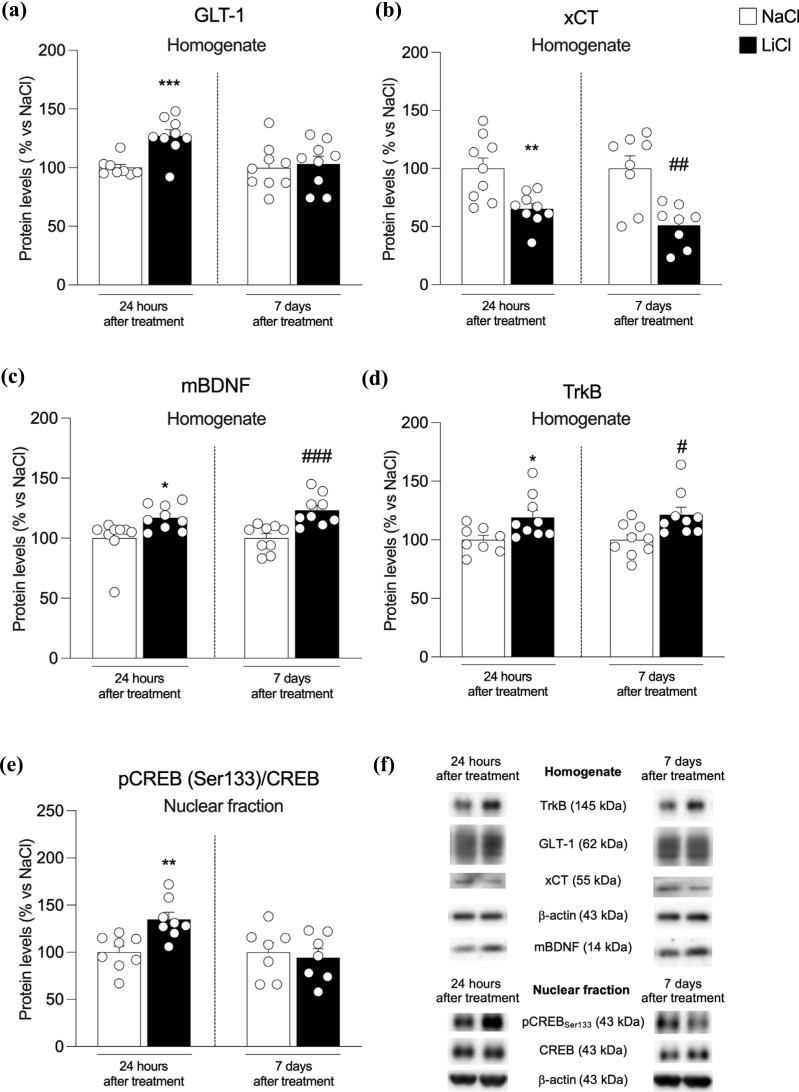
Protein expression levels of GLT-1 (panel **a**), xCT (panel **b**), mBDNF (panel **c**), TrkB (panel **d**) in the homogenate fraction and pCREB(Ser133)/CREB ratio, expressed as the ratio between the phosphorylated and the total form of the protein, (panel **e**) in the nuclear fraction from the hippocampus of rats 24 h and 7 days after the last NaCl and LiCl injection. In panel (**f**), representative immunoblots for GLT-1, xCT, mBDNF, TrkB, pCREB(Ser133), CREB, and β-actin are shown for each fraction evaluated. Results are expressed as mean percentage ± mean standard error. Unpaired Student’s t-test *p < 0.05, ***p < 0.01, ***p < 0.001 *vs.* NaCl 24 h; ^#^*p <* 0.05, ^##^*p <* 0.01, ^###^*p <* 0.001 *vs.* NaCl 7 d.

Since levels of postsynaptic NMDA receptors could be due, at least in part, to extracellular glutamate levels regulated by GLT-1, the main glial transporter of the neurotransmitter [22, 23]. We investigated that GLT-1 expression increased 24 hours after the exposure to LiCl (Fig. **2a**-**f**) while remaining unchanged 7 days later (Fig. **2a**: +27% *vs*. NaCl 24 h *p* = 0.0006, t(15) = 4.34 and +3% *vs*. NaCl 7 d, *p* = 0.75, t_(16)_ = 0.32) [22, 23]. Furthermore, we found that GLT-1 expression is increased 24 hours after the last exposure to LiCl while unchanged 7 days later (Fig. **2a**: +27% *vs.* NaCl 24 h *p* = 0.0006, t_(15)_ = 4.34 and +3% *vs.* NaCl 7 d, *p* = 0.75, t_(16)_ = 0.32).

We next examined the expression of the antiporter xCT, which exchanges intracellular glutamate with extracellular cystine, thus regulating glutamate extra-synaptic levels [23]. This antiporter expression is significantly reduced 24 hours and 7 days after the last administration (Fig. **2b**: -35% *vs.* NaCl 24 h *p* = 0.003, t_(16)_ = 3.38 and -49% *vs.* NaCl 7 d, *p* = 0.0018, t_(14)_ = 3.84).

It is known that Ca2+ entry through GluN2B-containing NMDA receptors shuts cyclic AMP response element binding protein (CREB) activation off [27]. Conversely, entry through GluN2A-containing NMDA receptors activates CREB, leading to the elevation of BDNF expression [41]. Therefore, we measured CREB phosphorylation and BDNF expression in the hippocampus of LiCl-treated rats 24 h and 7 days after the last exposure. CREB is activated at 24 hours while unchanged at the later time point (Fig. **2e**: +35% *vs.* NaCl 24 h *p* = 0.003, t_(14)_ = 3.53 and -6% *vs.* NaCl 7 d, *p* = 0.68, t_(12)_ = 0.41); whereas, at both time points, BDNF (Fig. **2c**: +17% *vs.* NaCl 24 h *p* = 0.02, t_(16)_ = 2.58 and +23% *vs.* NaCl 7 d, *p* = 0.0008, t_(16)_ = 4.11) and its high-affinity receptor TrkB (Fig. **2d**: +19% *vs.* NaCl 24 h *p* = 0.023, t_(15)_ = 2.54 and +22% *vs.* NaCl 7 d, *p* = 0.014, t_(16)_ = 2.75) expressions are increased.

**Fig. (3):**
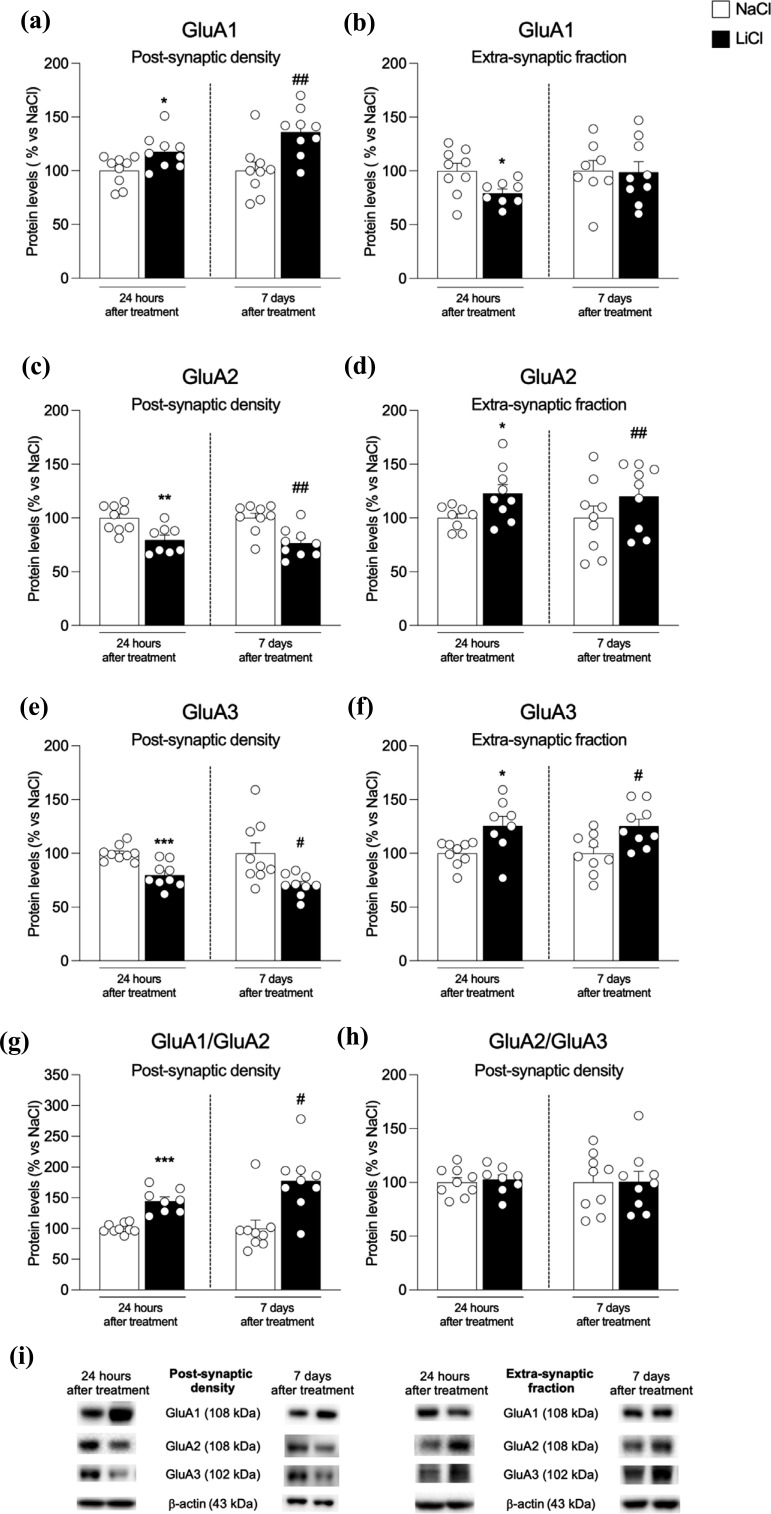
Protein expression levels of the main AMPA receptor subunits in the hippocampus of rats 24 h and 7 days after the last NaCl and LiCl injection. GluA1 (panel **a**, **b**), GluA2 (panel **c**, **d**), and GluA3 (panel **e**, **f**) levels were investigated in the postsynaptic density and the extra-synaptic fraction. The GluA1/GluA2 (panel **g**) and GluA2/GluA3 ratios (panel **h**) were measured in the postsynaptic density. In panel (**i**), representative immunoblots for GluA1, GluA2, GluA3, and β-Actin are shown for each fraction evaluated. Results are expressed as mean percentage ± mean standard error. Unpaired Student’s t-test or Mann-Whitney test *p < 0.05, **p < 0.01, ***p < 0.001 *vs.* NaCl 24 h; ^#^*p <* 0.05, ^##^*p <* 0.01, ^###^*p <* 0.001 *vs.* NaCl 7 d.

We next analyzed the expression and trafficking of AMPA receptors, which are critical for the action of LiCl [42]. GluA1 subunit expression is increased at both time points in the post-synaptic density (Fig. **3a**: +18% *vs.* NaCl 24 h p = 0.023, t_(16)_ = 2.50 and +36% *vs.* NaCl 7 d, *p* = 0.004, t_(16)_ = 3.31) whereas, at extra-synaptic sites, a reduction at 24 hours and unaltered state at 7 days were observed (Fig. **3b**: - 21% *vs.*NaCl 24 h*p*= 0.026, t_(15)_= 2.47 and -1%*vs.*NaCl 7 d,*p*= 0.91, t_(15)_= 0.11). Analysis of GluA2 and GluA3 AMPA subunits revealed a reduction in the post-synaptic density (Fig. **3c**: -20% *vs.* NaCl 24 h *p* = 0.004, U = 7 and -23% *vs.* NaCl 7 d, *p* = 0.0018, t_(16)_ = 3.74; Fig. **3e** -20% *vs.* NaCl 24 h *p* = 0.0005, t_(16)_ = 4.36 and - 29% *vs.* NaCl 7 d, *p* = 0.03, t_(16)_ = 2.38) while an up-regulation at extra-synaptic sites at both time points (Fig. **3d**: +23% *vs.* NaCl 24 h *p* = 0.033, t_(15)_ = 2.36 and +20% *vs.* NaCl 7 d, p = 0.004, t_(16)_ = 3.34; Fig. **3f**: +26% *vs.* NaCl 24 h *p* = 0.014, t_(15)_ = 2.77 and +25% *vs.* NaCl 7 d, *p* = 0.011, t_(16)_ = 2.87). We then measured GluA1/GluA2 and GluA2/GluA3 ratios to understand AMPA receptor subunit composition. We found that repeated administration of LiCl increased GluA1/GluA2 ratio in the hippocampus at both time points (Fig. **3g**: +45% *vs.* NaCl 24 h *p <* 0.0001, t_(15)_ = 6.45 and +77% *vs.* NaCl 7 d, *p* = 0.0106, U = 12) whereas no changes were observed in the GluA2/GluA3 ratio (Fig. **3h**: +2% *vs.* NaCl 24 h *p* = 0.71, t_(15)_ = 0.37 and +1% *vs.* NaCl 7 d, *p* = 0.96, t_(16)_ = 0.05).

**Fig. (4):**
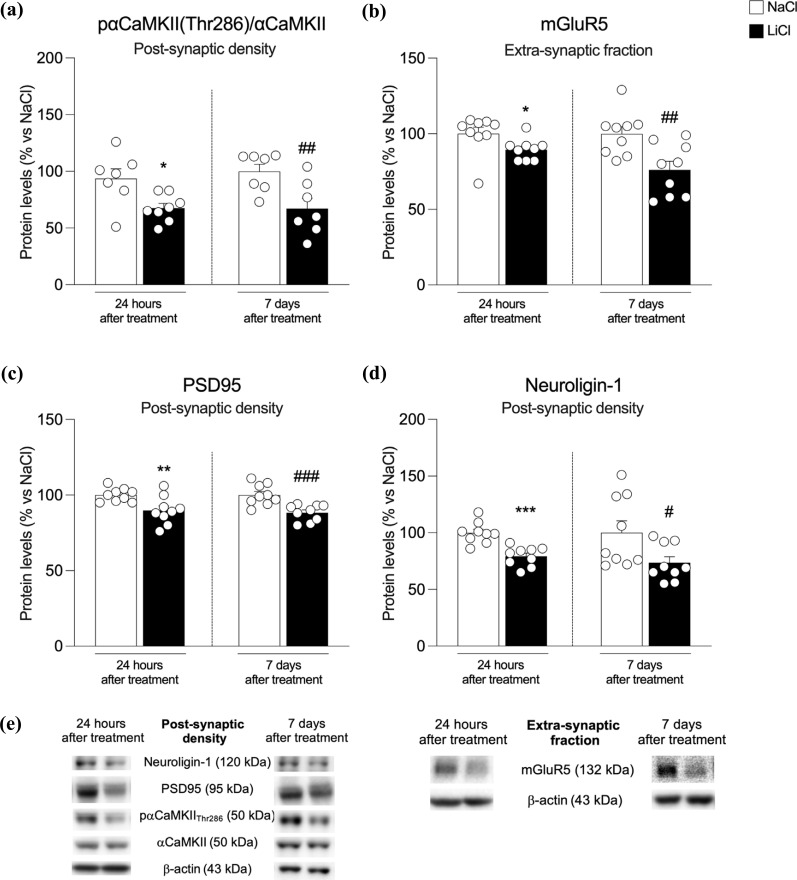
Protein expression levels of pαCαMKII(Thr286)/ αCαMKII ratio, expressed as the ratio between the phosphorylated and the total form of the protein, (panel **a**), mGluR5 (panel **b**), PSD95 (panel **c**), and Neurologin-1 (panel **d**) in the postsynaptic density from the hippocampus of rats 24 h and 7 days after the last NaCl and LiCl injection. In panel (**e**), representative immunoblots for pαCαMKII(Thr286), αCαMKII, mGluR5, PSD95, Neurologin-1, and β-actin are shown for each fraction evaluated. Results are expressed as mean percentage ± mean standard error. Unpaired Student’s t-test *p < 0.05, **p < 0.01, ***p < 0.001 *vs.* NaCl 24 h; ^#^*p <* 0.05, ^##^*p <* 0.01, ^###^*p <* 0.001 *vs.* NaCl 7 d.

Since the formation of GluA2-lacking Ca2+ permeable receptors may be excitotoxic by leading to a higher Ca2+ influx into the cell [43], we next examined the activation of αCαMKII. In the post-synaptic density of the hippocampus of chronically LiCl-exposed rats, αCαMKII autophosphorylation is significantly reduced at both time points (Fig. **4a**: -28% *vs.* NaCl 24 h *p* = 0.014, t_(13)_ = 2.82 and -33% *vs.* NaCl 7 d, *p* = 0.005, t_(14)_ = 3.29). Since the metabotropic glutamate receptor mGluR5 may limit Ca2+ influx into the cell [44], we measured mGluR5 at extra-synaptic sites where these receptors are mainly located [45]. Despite this fact that the receptor is expressed at sufficiently high levels on astrocytes [46], we observed that mGluR5 expression is reduced at both time points (Fig. **4b**: -11% *vs.* NaCl 24 h *p* = 0.044, t_(16)_ = 2.18 and -24% *vs.* NaCl 7 d, *p* = 0.005, t_(16)_ = 3.19) respectively.

Next, to investigate potential structural changes induced by repeated exposure to LiCl, we evaluated the expression of the structural protein PSD95 and Neuroligin-1. Our results show that PSD95 expression is reduced in the post-synaptic density at both time points (Fig. **4c**: -28% *vs.* NaCl 24 h *p* = 0.014, t_(16)_ = 2.82 and -33% *vs.* NaCl 7 d, *p* = 0.005, t_(16)_ = 3.29).

Analysis of Neuroligin-1, an adhesion protein that sticks together the glutamatergic presynaptic and postsynaptic terminals, revealed a similar reduction at both time points (Fig. **4d**: -28% *vs.* NaCl 24 h *p* = 0.014, t_(16)_ = 2.82 and -33% *vs.* NaCl 7 d, *p* = 0.005, t_(16)_ = 3.29).

**Fig. (5):**
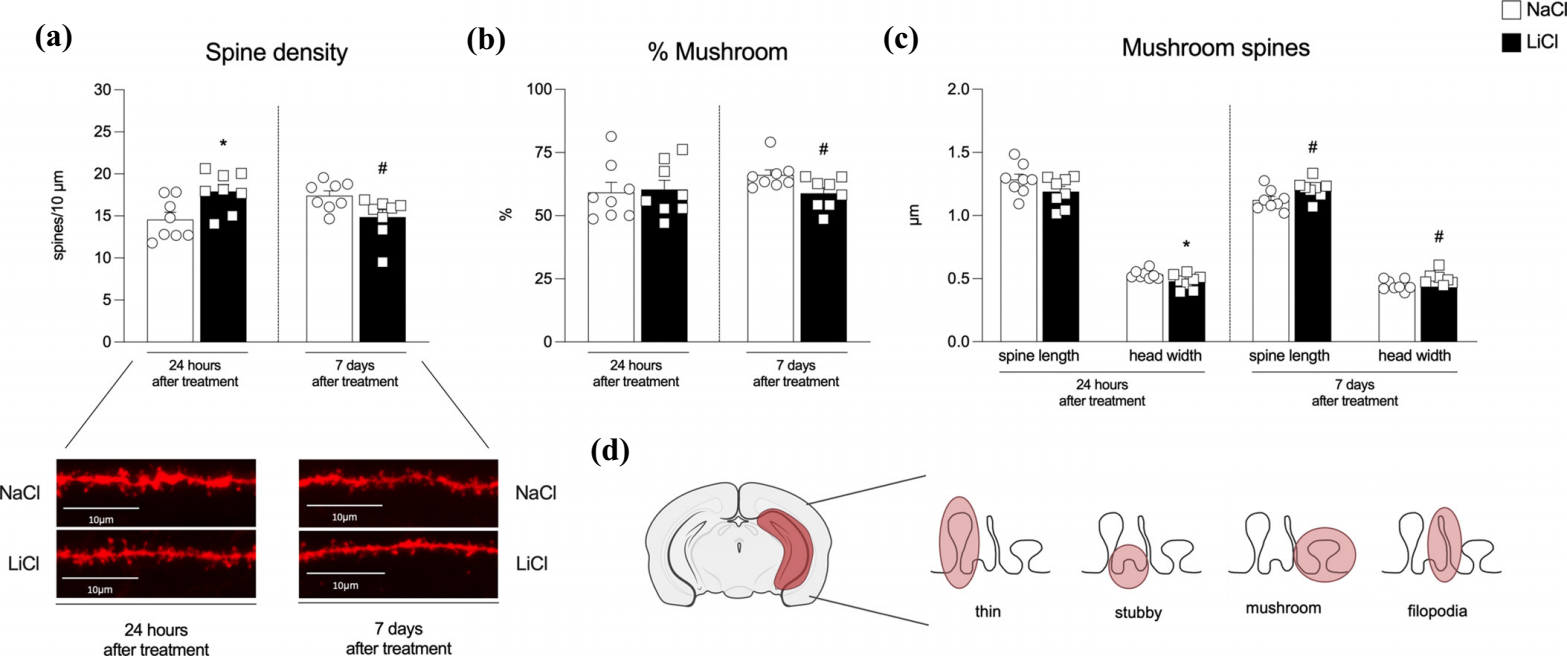
Effects on dendritic spine density and morphology in the hippocampus of rats 24 hours or 7 days after the last NaCl or LiCl treatment. Panel (**a**) shows the total spine density in the hippocampus and, underneath, representative pictures from each experimental group. The percentage of mushroom-shaped spines and their relative length and head width, for each time point considered, are represented in panel (**b**) and panel (**c**), respectively. n > 1800 spines from at least 20 different neurons for each group, neurons analyzed belonged to 8 hemispheres per group. Results are expressed as mean percentage ± mean standard error. Unpaired Student’s t-test or Mann-Whitney test *p < 0.05 *vs.* NaCl 24 h; #*p <* 0.05 *vs.* NaCl 7 d. Panel (**d**) shows a schematic representation of the brain area considered, the entire hippocampus, and an example of spine shapes (thin, stubby, mushroom and filopodia).

Since we observed alterations in PSD95 protein levels at 24 hours and 7 days after the last LiCl treatment, we supposed a remodeling in hippocampal dendritic spines. Accordingly, using a dualistic labeling technique, we analyzed hippocampal dendritic spine density and morphology (Fig. **5a**-**d**). We found an overall increase in spine density levels 24 hours after the last LiCl exposure (Fig. **5a**: *p* = 0.014, t_(14)_ = 2.78) and a reduction 7 days later (Fig. **5a**: *p* = 0.028, U = 11). Further, when classifying the shape of all spines into the main types (mushroom, thin and stubby), we found a reduction in the percentage of mushroom-shaped spines, *i.e*., the active and mature spines, only 7 days after the last LiCl treatment (Fig. **5b**: +1.18% *vs.* NaCl 24 h *p* = 0.83, t_(14)_ = 0.22 and -7.19% *vs.* NaCl 7 d *p* = 0.029, t_(14)_ = 2.43). No changes were observed in the percentages of stubby- and thin-shaped spines (Supplementary Table **1a**). A deeper analysis of mushroom-shaped spines revealed no variations in spine length 24 hours after LiCl treatment (Fig. **5b**: *p* = 0.13, t_(14)_ = 1.59), while we observed a reduction in head width size (Fig. **5b**: *p* = 0.038, t = 2.28). On the contrary, we observed an increase in both spine length and head width 7 days after the last LiCl treatment (Fig. **5b**: *p* = 0.04, t_(14)_ = 2.25; *p* = 0.044, t_(14)_ = 2.21, respectively). No changes were observed in the length and head width of stubby- and thin-shaped spines (Supplementary Table **1b**).

## 4 DISCUSSION

**Fig. (6):**
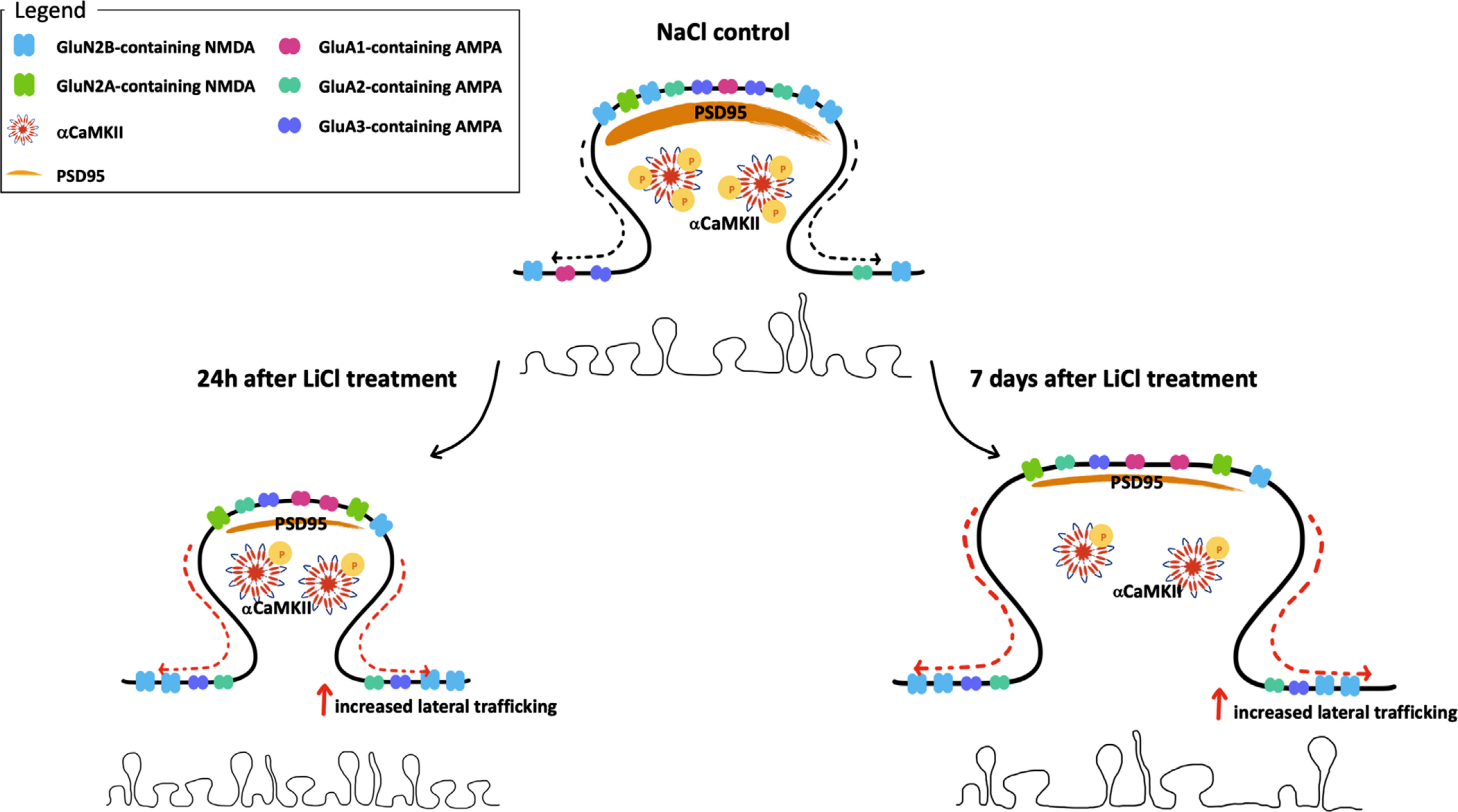
Schematic representation of the changes observed in the hippocampal synapse of LiCl-treated rats 24 h and 7 days after the last injection. *AMPA*, glutamate α-amino-3-hydroxy-5-methyl-4-isoxazole propionic acid receptors; *NMDA*, N-methyl-D-aspartate receptors; *PSD95* postsynaptic density protein 95; αCαMKII, Ca^2+^/CaM-dependent protein kinase II type α.

Our findings illustrate a complex chain of interconnecting changes/adaptations of glutamate homeostasis set in motion by repeated exposure to chronic LiCl treatment (Fig. **6**), an observation in line with the evidence that LiCl does not exert its antimanic effect through a single but instead, *via* multiple mechanisms.

Regarding the homeostasis of glutamatergic neurotransmission as a central mechanism, the complexity of the action of LiCl can be broken down by analyzing the different pieces of the puzzle individually. At first sight, the analysis of LiCl-induced effects on NMDA receptors reveals a peculiar pattern of regulation as the expression of the mandatory subunit GluN1 and its accessory subunit GluN2B are reduced at a post-synaptic level while increased at extra-synaptic sites. The post-synaptic reduction of these subunits may contribute to toning down glutamatergic inputs and promote the antimanic action of LiCl, in agreement with the widely accepted theory that BD is characterized by increased glutamatergic tone [12, 13]. However, increased trafficking toward extra-synaptic sites of GluN2B-containing NMDA receptors may activate pro-apoptotic mechanisms [41]. Of note, together with such increased trafficking of GluN2B-containing NMDA receptors at extra-synaptic sites, the repeated treatment with LiCl has also promoted some specific defensive strategies.

In fact, since extra-synaptic NMDA receptors are usually activated by glutamate spillover, it appears that such effect can be counterbalanced at 24 h by the increased expression of GLT-1, which promotes the reuptake of extracellular glutamate into astrocytes, but also by the reduced expression of the antiporter xCT, which secretes glutamate from, and imports cystine into, the glial cell, contributing to lowering glutamate spillover at extra-synaptic sites [23] and preventing the activation of extra-synaptic NMDA receptors. However, one week later, the mechanisms promoting GLT-1 up-regulation are likely exhausted and may no longer buffer against glutamate spillover. Interestingly, our data point out that repeated administration of LiCl has caused a marked reduction of xCT expression also at 7 days post-injection indicating the persistence of reduced glutamate spillover through the antiporter, a mechanism that may intriguingly be operative in patients to reduce glutamate hyperexcitability over time. Notably, it has been observed that in the hippocampus of xCT mutant mice, extracellular glutamate concentrations are reduced by 60%, and no cell death was detected [47].

Besides highlighting some preventative strategies set in motion by repeated administration of LiCl, our data also highlight the hypothesis that LiCl-induced extra-synaptic trafficking of GluN1-GluN2B receptors is not toxic for the cell. In fact, since it is clear that Ca^2+^ entry *via* GluN2B- containing NMDA receptors dampens CREB activation [48], the evidence that CREB phosphorylation is significantly increased at 24 h and unaltered at 7 days suggests that there is most like no LiCl-induced glutamate spillover with activation of GluN2B-containing NMDA receptors. Conversely, since it is well established that Ca^2+^ entry through GluN2A- containing NMDA receptors activates CREB, thus promoting BDNF synthesis [41], our results showing increased GluN2A and BDNF expression suggest that repeated exposure to LiCl primarily activates GluN2A-containing NMDA receptors thus exerting neuroprotection. In addition, it has been demonstrated that the activation of GluN2A-containing receptors counteracts GluN2B-containing receptor-mediated cell death by promoting pro-survival signaling [32]. Further, activation of GluN2A subunit-mediated signaling favors the maturation and plasticity of the glutamatergic synapse [39]. This is supported by the increased GluN2A/GluN2B ratio, which is indicative of reduced vulnerability as a consequence of the formation of synapses characterized by an increased number of functional contacts [49]. Taken together, these data have highlighted a series of mechanisms that come into play to boost the neuroprotective action of LiCl and limit its potentially toxic effects.

In line with the situation depicted examining NMDA receptor-mediated homeostasis, also the LiCl-induced expression and trafficking of AMPA receptors reflect a complex regulation. In fact, on one side, we observed that the expression of GluA2 is significantly reduced in the post-synaptic density, leading to GluA2-lacking AMPA receptors, whose formation may be excitotoxic, by driving a higher influx of Ca^2+^ in the cell. However, in concomitance, we also observed a reduction of αCαMKII autophosphorylation at Thr286, indicative of reduced intracellular Ca^2+^ levels. The increased formation of GluA2-lacking AMPA receptors may be buffered by limiting Ca^2+^ release from intracellular stores, an effect that might be driven by the reduced expression of mGluR5, as previously suggested [44].

In addition, the reduced expression of Neuroligin-1, indicative of a less stable connection between pre- and post-synaptic glutamatergic sites, may contribute to altering synaptic communication, thus rendering less efficient the increased number of GluA2-lacking AMPA receptors. Further, the increase of GluA2-lacking AMPA receptors may also be compensated by the up-regulation of GluA1 expression. Previous data have established that a LiCl- induced increase of synaptic levels of GluA1 is associated with improved mania- and depression-related behaviors in mice [20] and that overexpression of GluA1 AMPA receptors normalizes mania-like behavior in mice [50]. Of note, such an increase could be mediated by the increased expression of BDNF, known to modulate the expression of the GluA1 AMPA receptor subunit during synaptic function regulation [51]. Thus, the action of LiCl can be ascribed to the combined action/synergy of different mechanisms that, all together, promote a wave of neuroplasticity in the brain, as here suggested by recruiting the GluA1 AMPA receptor subunit and the neurotrophin BDNF, evincing that synaptic, intracellular, and neuroprotective mechanisms combine to boost hippocampal neuroplasticity following repeated LiCl exposure. The evidence that the BDNF-GluA1 pathway, together with GluN2A-containing NMDA receptors, are increased as early as 24 h but also persist 7 days after the last LiCl injection suggests that such mechanisms might represent an initial step leading to the reorganization of the glutamatergic synapses that could ultimately sustain the long-term antimanic effects of LiCl, further in the hippocampus of LiCl-treated rats.

Changes in the levels of synaptic proteins and signaling molecules have been shown to influence the density and morphology of the dendritic spines and, consequently, synaptic functions [52]. Accordingly, we decided to investigate spine morphology in the hippocampus of LiCl- treated rats. Our results identify changes in dendritic spine morphology. In fact, it appears that 24 hours after the last LiCl exposure, spine density is increased; conversely, one week later, we found a marked reduction in spine density. Nonetheless, although the percentage of mushroom-shaped spines, *i.e*., the most active type of dendritic spines [53], is reduced 7 days following the last exposure, their neck length and head width are enhanced. Since spine head volume has been positively associated with synaptic function [54], we can argue that at 24 hours post-LiCl treatment the reduced head width of mushroom-shaped spines suggests a decreased general excitability of mature spines, an observation in line also with the reduced expression of PSD95. Conversely, a week later, the most intriguing observation is, indeed, the reduced percentage of mushroom-shaped spines, an effect that may be indicative of reduced communicative synapses and their strengthening. However, on the contrary, the evidence of increased length and head diameter of such spines calls, instead, for adaptive mechanisms set in motion to sustain hippocampal synaptic communication.

We are aware that our study holds some limitations. First, we did not administer LiCl in an animal model of BD; however, we also know how difficult it is to recapitulate in an animal model the cyclicity between mania and depression, typical of BD patients. Importantly, our study was undertaken in male rats; therefore, we do not know whether the results can be extended to female rats. Further, we limited our analyses to the hippocampus, and we cannot rule out that the glutamatergic picture may be different in other brain regions; also, since preparing enriched post-synaptic and extra-synaptic fractions requires a certain amount of brain tissue, we investigated the whole hippocampus without subdividing it into the ventral and the dorsal portions, which are known to modulate different functions. Finally, we cannot determine whether the changes in glutamatergic markers shown here are due to the last injection of LiCl or the entire history of LiCl exposure.

## CONCLUSION

In conclusion, repeated exposure to LiCl acts through multiple, parallel, but also converging forms of compensatory neuroplasticity, highlighting an overall mechanism through which it may be effective in BD. Within such a complex set of molecular and structural analyses, we observed a profound and persistent reorganization of the glutamatergic postsynaptic density composition and structure. We, thus, hypothesize that the herein-shown hippocampal effect of long-term exposure to LiCl may be globally beneficial as it may be the result of positive actions represented by the rearrangement of synaptic NMDA and AMPA receptors coupled with a potentiation of the plasticity-promoting action induced by increased GluN2A expression, up-regulation of BDNF signaling and GluA1 expression, all combined with morphological rearrangements indicative of a more plastic synapse.

## Data Availability

The data that support the findings of this study is available from the corresponding authors on a reasonable request.

## LIST OF ABBREVIATIONS

BD: Bipolar Disorder
GLT-1: Glutamate Transporter
LiCl: Lithium Chloride
PBS: Phosphate Buffer
PSD95: Postsynaptic Density Protein 95
