# Supplementary material for: Chronic Lithium Treatment Alters NMDA and AMPA Receptor Synaptic Availability and Dendritic Spine Organization in the Rat Hippocampus
Source: Curr Neuropharmacol. 2023 Sep 15;22(12):2045–58. doi: 10.2174/1570159X21666230913144420 (PMC11333793; doi:10.2174/1570159X21666230913144420)
Supplement: Supplementary file 1 [file CN-22-2045_SD1.pdf]

Supplementary Material

Chronic Lithium Treatment Alters NMDA and AMPA Receptor Synaptic Availability and Dendritic Spine Organization in the Rat Hippocampus

Lucia Caffino[1,#], Giorgia Targa[1,#], Anne Stephanie Mallien[2], Francesca Mottarlini[1], Beatrice Rizzi[1], Judith R. Homberg[3], Peter Gass[2,°] and Fabio Fumagalli[1,°,*]

[1]*Department of Pharmacological and Biomolecular Sciences 'Rodolfo Paoletti', Università degli Studi di Milano, Via Balzaretti 9, 20133, Milan, Italy;* [2]*Department of Psychiatry and Psychotherapy, RG Animal Models in Psychiatry, Central Institute of Mental Health, Medical Faculty Mannheim, Heidelberg University, Mannheim, Germany;* [3]*Department of Cognitive Neuroscience, Division of Molecular Neurogenetics, Donders Institute for Brain, Cognition and Behaviour, Radboud University Nijmegen Medical Centre, The Netherlands*

Supplementary figure 1. Cropped immunoblot related to the expression levels of GluN2A, GluN2B, GluN1, Neuroligin-1, GluA1, GluA2, PSD95, $pCaMKII_{Thr286}$, CaMKII, β-actin measured in the **post-synaptic density** of the Hippocampus of rats sacrificed 24 h after the last injection of NaCl or LiCl, presented in figures 1, 3, 4.

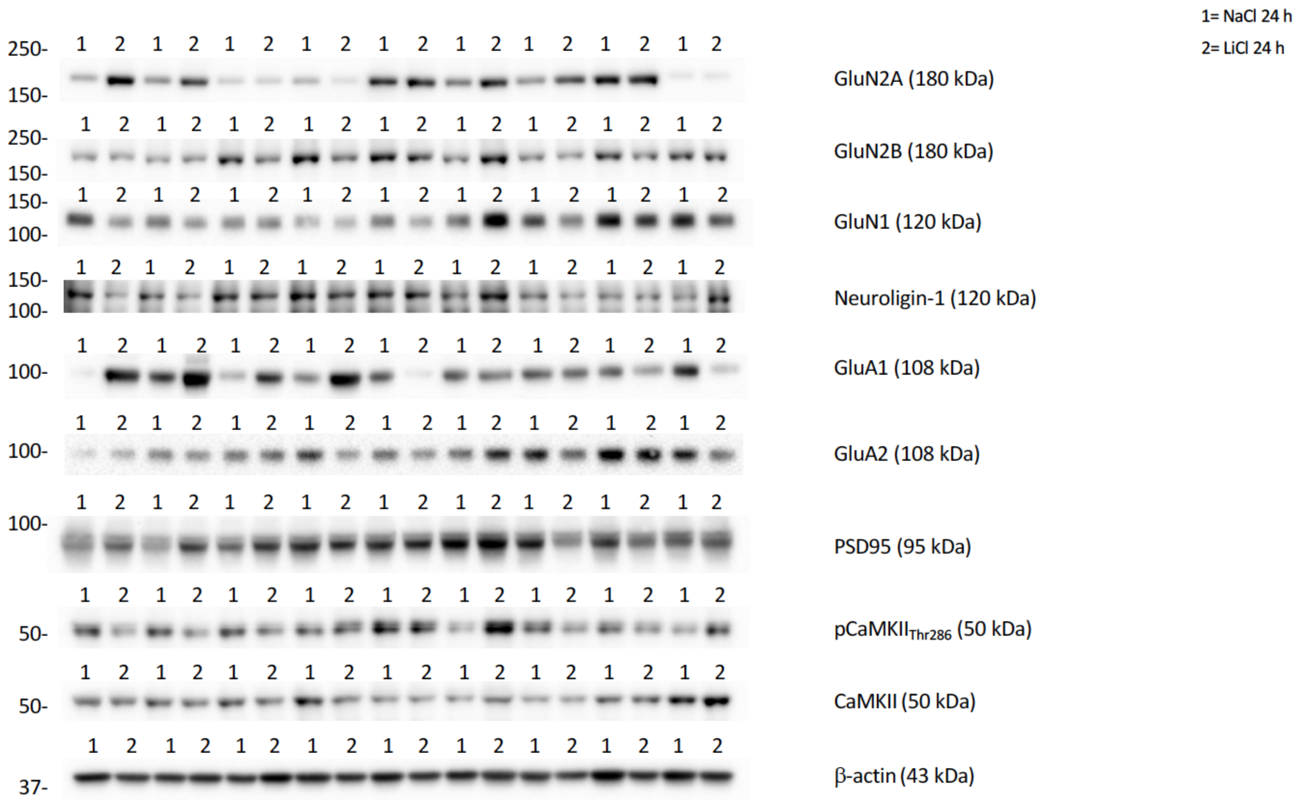

1875-6190/24 $65.00+.00 © 2024 Bentham Science Publishers

Supplementary figure 2. Cropped immunoblot related to the expression levels of GluN2A, GluN2B, GluN1, Neuroligin-1, GluA1, GluA2, PSD95, pCaMKII$_{Thr286}$, CaMKII, β-actin measured in **the post-synaptic density** of the Hippocampus of rats sacrificed 24 hours and 7 days after the last injection of NaCl or LiCl, presented in figures 1, 3, 4.

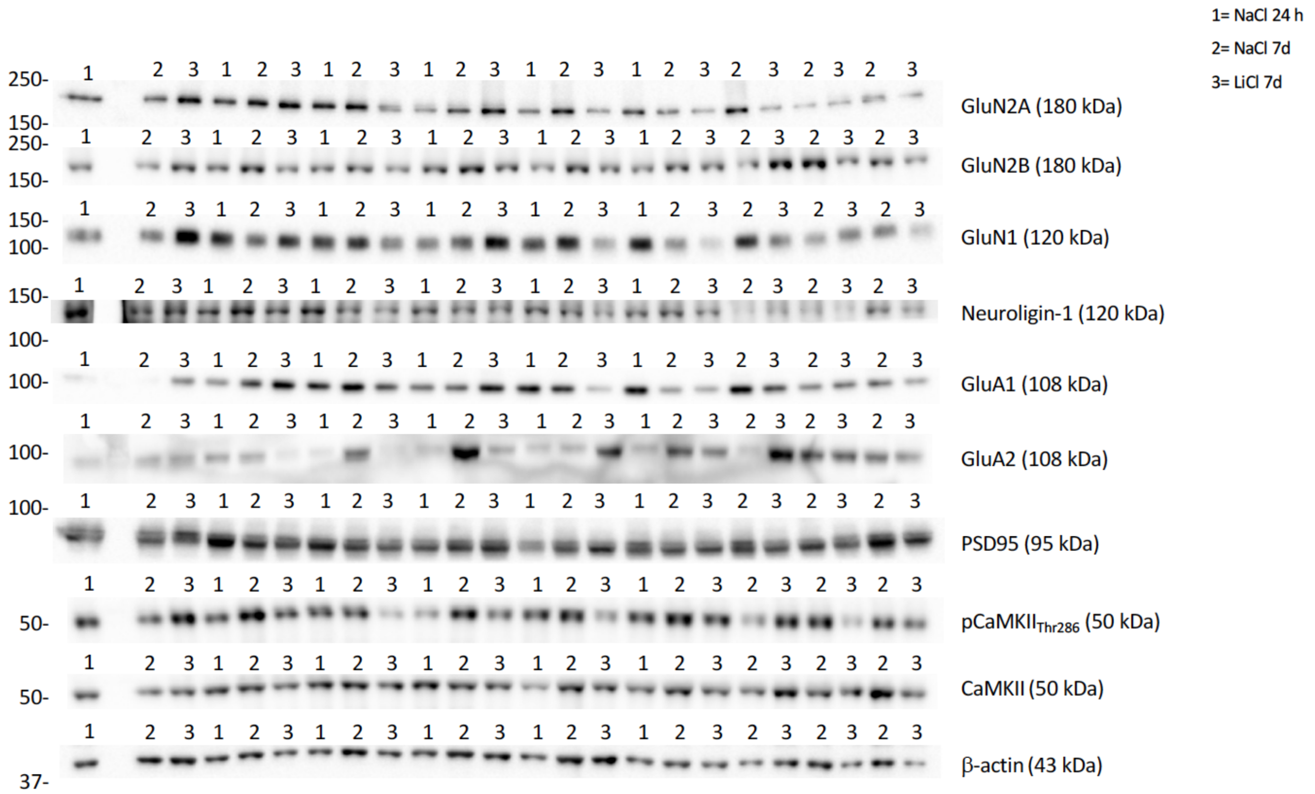

Supplementary figure 3. Cropped immunoblot related to the expression levels of GluA3 and β-actin measured in the **post-synaptic density** of the Hippocampus of rats sacrificed 24 hours (a) and 7 days (b) after the last injection of NaCl or LiCl, presented in figure 3.

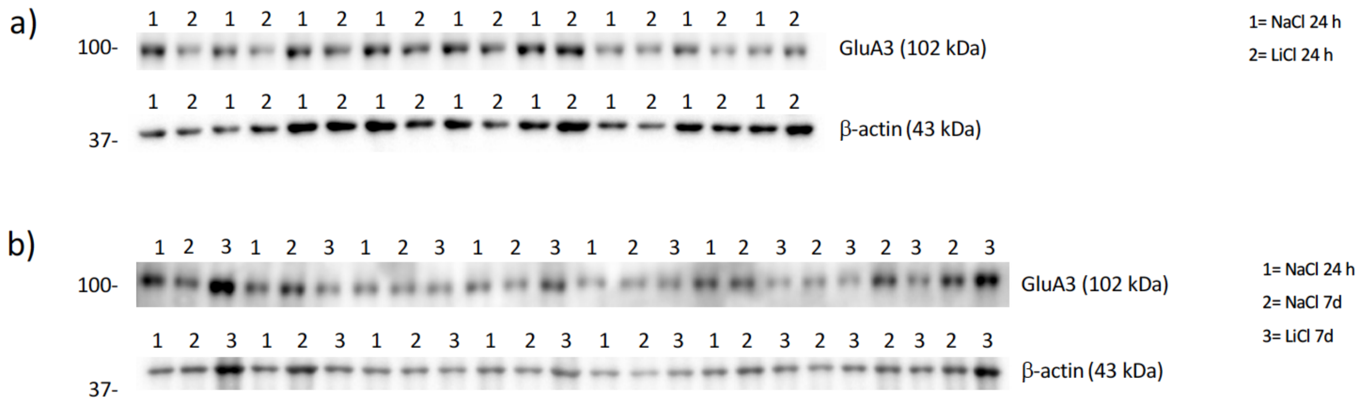

Supplementary figure 4. Cropped immunoblot related to the expression levels of GluN2B, mGluR5, GluN1, GluA1, GluA2, GluA3, β-actin measured in the **extra-synaptic fraction** of Hippocampus of rats sacrificed 24 h after the last injection of NaCl or LiCl, presented in figures 1, 3, 4.

1= NaCl 24h

2= LiCl 24h

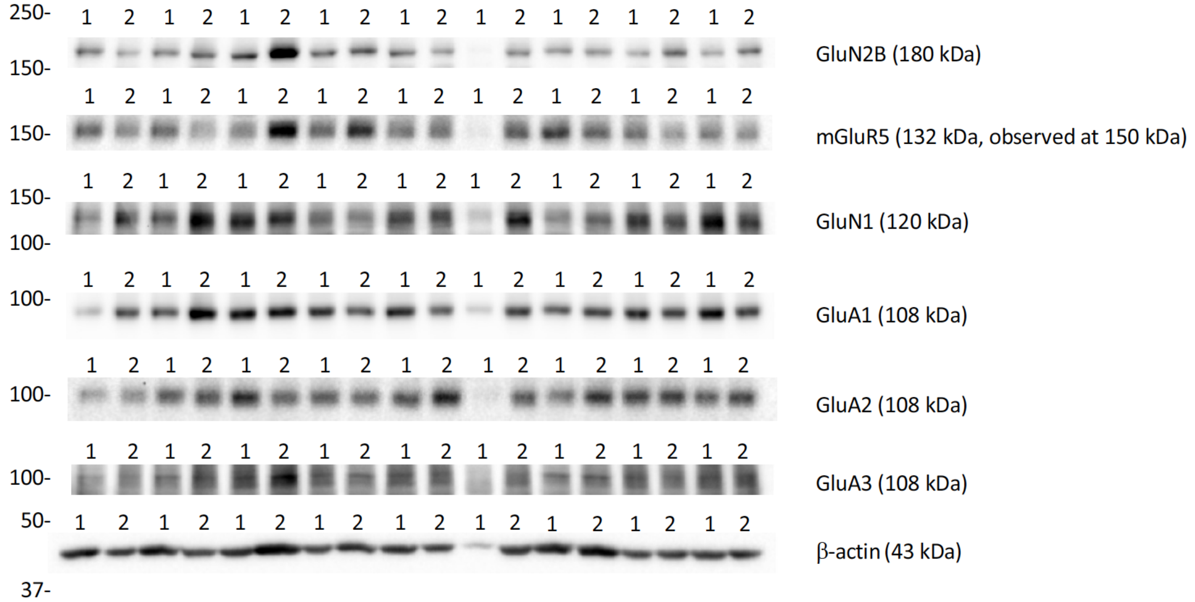

Supplementary figure 5. Cropped immunoblot related to the expression levels of GluN2B, mGluR5, GluN1, GluA1, GluA2, GluA3, β-actin measured in the **extra-synaptic fraction** of Hippocampus of rats sacrificed 24 and 7 days after the last injection of NaCl or LiCl, presented in figures 1, 3, 4

1= NaCl 24 h

2= NaCl 7d

3= LiCl 7d

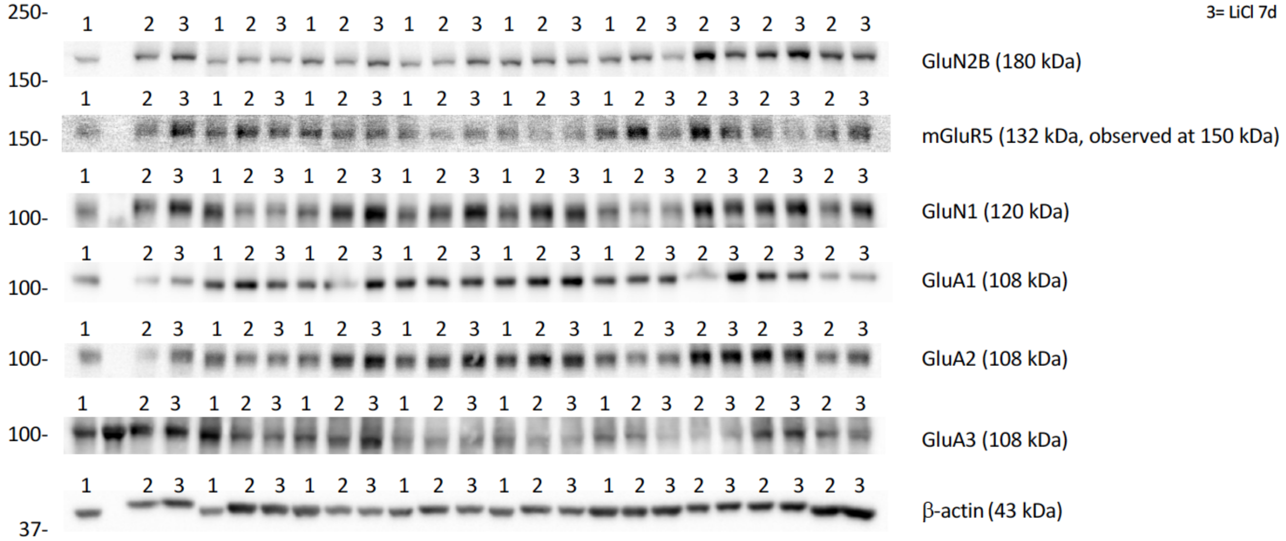

Supplementary figure 6. Cropped immunoblot related to the expression levels of TrkB, GLT-1, xCT, β-actin, and mBDNF measured in the **homogenate** of the Hippocampus of rats sacrificed 24 hours (a) and 7 days (b) after the last injection of NaCl or LiCl, presented in figure 2.

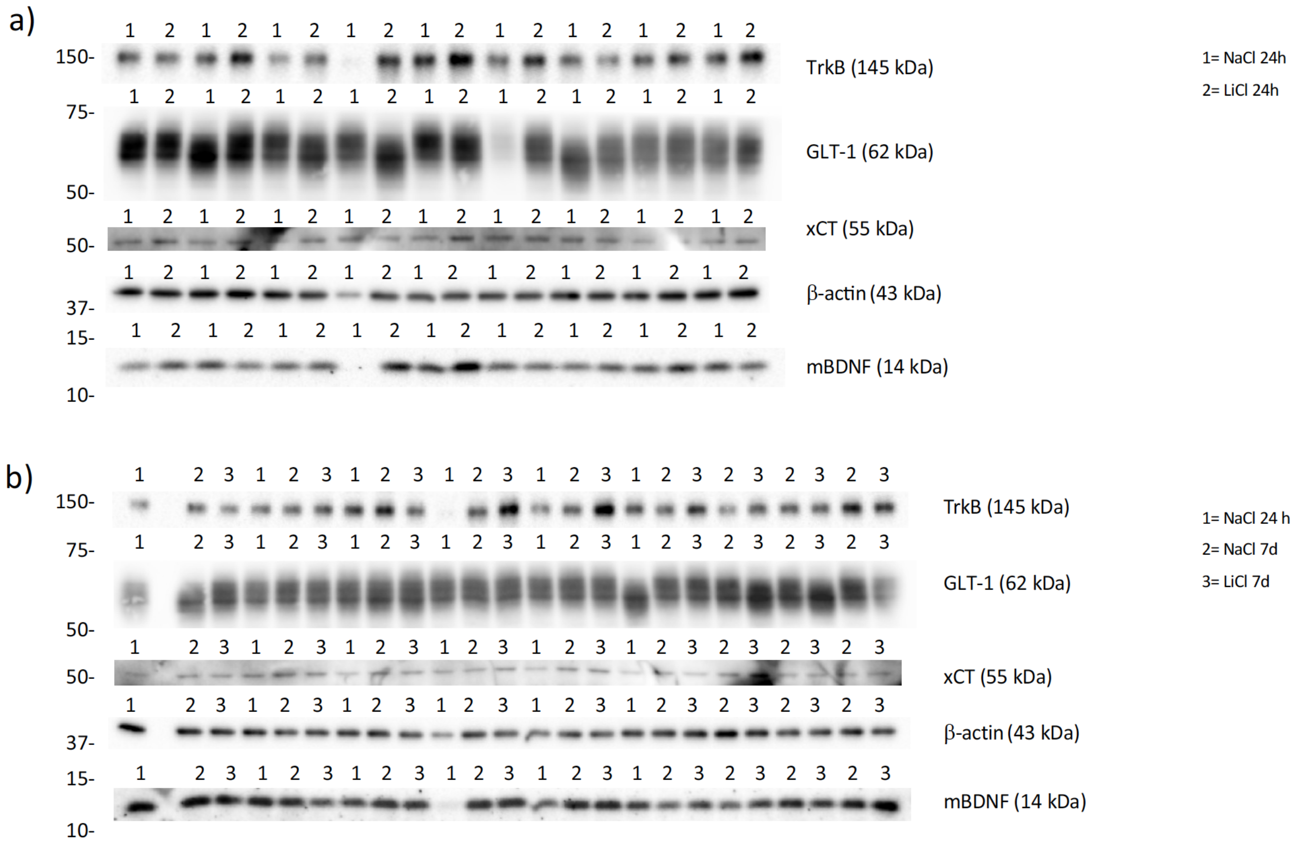

Supplementary figure 7. Cropped immunoblot related to the expression levels of pCREB$_{Ser133}$, CREB, and β-actin measured in the **nuclear fraction** of the Hippocampus of rats sacrificed 24 hours (a) and 7 days (b) after the last injection of NaCl or LiCl, presented in figure 2.

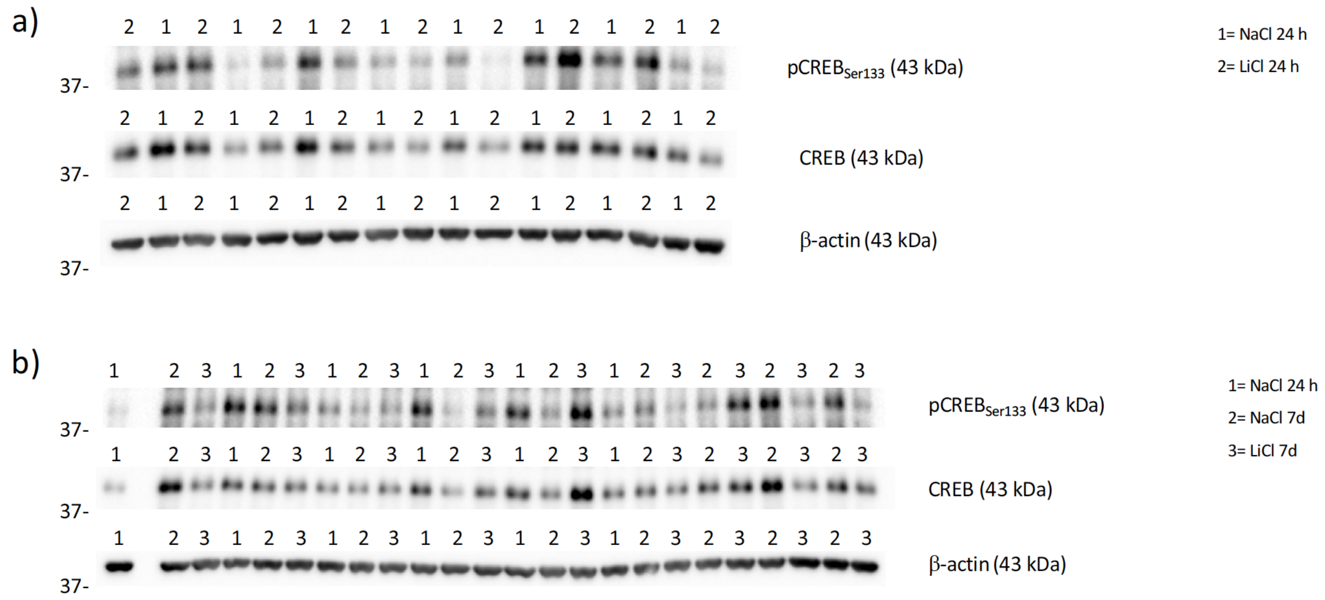

Supplementary Table 1. Effects of LiCl treatment on dendritic spine morphology in the hippocampus of rats sacrificed 24 hours or 7 days after the last exposure. Table (a) shows the percentage of thin-, stubby-shaped spines and filopodia, expressed as mean percentage ± mean standard error. Table (b) shows the length and head width of thin- and stubby-shaped spines, expressed as μm± mean standard error.

a)

	**% Thin**		**% Stubby**		**% filopodia**	
	NaCl	LiCl	NaCl	LiCl	NaCl	LiCl
24 hours after the last treatment	13,63 ± 3,71	13,70 ± 4,03	21,03 ± 2,80	21,09 ± 2,22	6,14 ± 2,68	4,82 ± 2,09
7 days after the last treatment	7,45 ± 1,98	12,22 ± 3,50	22,31 ± 1,94	21,72 ± 1,24	6,22 ± 1,84	4,74 ± 2,06

b)

	**Thin (μm)**				**Stubby (μm)**			
	NaCl		LiCl		NaCl		LiCl	
	Spine length	Head width	Spine length	Head width	Spine length	Head width	Spine length	Head width
24 hours after the last treatment	1,27 ± 0,04	0,51 ± 0,05	1,19 ± 0,07	0,43 ± 0,02	0,80 ± 0,01	0,41 ± 0,02	0,79 ± 0,01	0,37 ± 0,02
7 days after the last treatment	1,25 ± 0,02	0,57 ± 0,09	1,27 ± 0,05	0,53 ± 0,03	0,76 ± 0,01	0,36 ± 0,01	0,78 ± 0,01	0,37 ± 0,01
